# A National Initiative to Support Internationally Educated Nurses: Implementation and Policy Insights from the PNAA Cy Pres Program

**DOI:** 10.3390/healthcare14121742

**Published:** 2026-06-17

**Authors:** Mary Joy Garcia-Dia, Reynaldo R. Rivera, Maria Luisa B. Ramira, Marife Sevilla, Lolita B. Compas, Laarni C. Florencio, Madelyn D. Yu, Lorraine S. Evangelista

**Affiliations:** 1 Frances Payne Bolton School of Nursing, Case Western Reserve University, Cleveland, OH 44106, USA; 2 NewYork-Presbyterian Hospital, New York, NY 10065, USA; rrr9001@nyp.org (R.R.R.); lcf9006@nyp.org (L.C.F.); 3 School of Nursing, Columbia University, New York, NY 10032, USA; 4 College of Nursing and Health Sciences, United States University, San Diego, CA 92108, USA; mramirapnasd2014@gmail.com; 5 Philippine Nurses Association of America, North Brunswick, NJ 08902, USA; marife.sevilla@gmail.com (M.S.); lolitabcompas@aol.com (L.B.C.); pnaapres1820@gmail.com (M.D.Y.); 6 Sue and Bill Gross School of Nursing, University of California, Irvine, CA 92697, USA; evangell@hs.uci.edu

**Keywords:** internationally educated nurses, nurse migration, workforce integration, ethical recruitment, healthcare workforce, organizational governance, stakeholder engagement, nursing workforce policy, implementation science

## Abstract

**Background**: The integration of internationally educated nurses (IENs) into healthcare workforces is expanding globally, yet organization-led support models remain understudied. Successful IEN integration requires ethical recruitment, structured onboarding, workforce support, and stakeholder engagement in policy discussions related to transition and retention. **Objective**: To examine the conceptualization, implementation, and policy implications of the Philippine Nurses Association of America Cy Pres Task Force’s national initiative to support IEN onboarding and transition into U.S. healthcare. **Methods**: This descriptive program evaluation utilized governance documents, program planning records, policy summit materials, aggregated survey findings, PNAA Human Rights Committee resources, and the *Handbook for Filipino Nurses Immigrating to the United States* to examine initiative development, implementation processes, and program outputs. A descriptive narrative synthesis was used to characterize program structure, stakeholder engagement, and policy priorities. **Findings**: The PNAA Cy Pres governance model was built around ethical recruiting, workforce integration, and advocacy. The work began with policy summits with nurse leaders, health care organizations, recruitment agencies, and policy experts, focusing on hiring, onboarding, legal issues, and staff retention. Stakeholder engagement, interdisciplinary collaboration, and appreciative inquiry were used to identify best practices and goals. Key outputs included the establishment of a national governance structure, implementation of national and regional policy summits, and identification of policy priorities related to ethical recruitment, onboarding, workforce integration, and governance. **Conclusions**: The PNAA Cy Pres initiative provides an implementation-informed approach that may help guide future workforce integration efforts. The study illustrates how ethical recruitment, workforce integration, and stakeholder engagement can help translate workforce policy principles into practice. **Policy & Practice Implications**: Healthcare institutions, policymakers, and professional organizations need to work together to standardize onboarding, ethical recruitment, and support mechanisms to facilitate the integration and sustainability of the IEN workforce.

## 1. Introduction

### 1.1. Background

The international migration of nurses is increasing. Many nations are suffering from nursing shortages. Internationally educated nurses (IENs) are a key source of supply to satisfy the needs of health care organizations, particularly in the United States and other high-income nations. IENs bring professional expertise and cultural diversity, enhancing patient care. The Philippines has been a leading source country of nurses working abroad for decades, and Filipino nurses remain a key component of healthcare systems in the United States and globally [1,2,3,4].

International recruitment also presents key problems about fairness, transparency, and support. The World Health Organization’s Global Code of Practice on the International Recruitment of Health Personnel asks for ethical recruitment and protection of migrant health workers [5]. However, these rules are not uniformly enforced across countries, corporations, or recruitment agencies [6]. Recent studies continue to raise concerns about how IENs are recruited and supported. Concerns about unclear contracts, recruitment costs, power imbalances between nurses and employers/agencies, and the need for increased protection of nurses during recruitment and transition have been raised [7,8,9].

Many IENs are not new nurses. Although they often arrive with strong education and years of clinical experience, working in a new healthcare system presents challenges. For instance, IENs are required to meet licensure and credentialing standards, adapt to new workplace expectations, learn new ways of communicating, and understand the structure of care in the United States [10,11,12]. Tan and Alpert (2013) [13] were the first researchers to identify the learning needs of Filipino IENs, including orientation, continuing education, and better preparation for practice in the United States. Further research underscores the need for structured onboarding, mentorship, and culturally relevant support [14,15].

Fragmented support structures can make transition uneven and unnecessarily difficult. The level of onboarding and transition support varies widely; some nurses receive comprehensive assistance, while others receive very little. Key aspects of the transition process are often managed by separate groups, including recruitment, legal guidance, onboarding, mentorship, and workplace integration. When these groups do not coordinate effectively, gaps emerge that make the transition harder than necessary.

Professional nursing groups can work to overcome some of these challenges. They may rally with nurses, employers, recruiters, legal and policy experts, and nursing leaders. They can also help identify common challenges, share best practices, and support more ethical and coordinated efforts for IEN transition.

The Philippine Nurses Association of America (PNAA) established the Cy Pres Task Force in 2024 to help IENs enter and integrate into the US healthcare workforce. The effort included a national task force, stakeholder engagement, local conferences, and regional and national policy summits to address ethical recruitment, onboarding, legal issues, transition support, and retention. From a policy and health services perspective, this work is important because recruitment practices, licensure requirements, employer responsibilities, and workforce needs all influence how IENs enter and adapt to the U.S. healthcare workforce.

Although more attention is being given to IEN transition, fewer papers describe how professional nursing organizations can put ethical recruitment and workforce integration principles into action. This paper addresses that gap by describing the development, implementation, and policy relevance of the PNAA Cy Pres initiative as an organization-led effort to support IEN transition and workforce integration.

### 1.2. Purpose of the Initiative

The purpose of this paper is to describe the development and implementation of the PNAA Cy Pres initiative and to discuss its relevance for IEN workforce integration and policy. Specifically, this paper describes: (1) the structure and governance of the initiative; (2) key program activities, including policy summits and stakeholder engagement; and (3) lessons learned that may inform future efforts to support IEN recruitment, onboarding, transition, and retention. By presenting this initiative, we aim to show how a professional nursing organization can help translate ethical recruitment and workforce integration principles into coordinated action.

## 2. Materials and Methods

### 2.1. Project Design

This paper used a descriptive program evaluation approach to examine the development and implementation of the PNAA Cy Pres initiative. The aim was not to test an intervention or measure program effectiveness. Instead, the evaluation focused on describing how the initiative was organized, what activities were implemented, what outputs were produced, and what lessons may inform future IEN workforce integration efforts.

### 2.2. Program Description

The PNAA Cy Pres initiative was developed through the PNAA Human Rights Committee to support internationally educated nurses entering and transitioning into the U.S. healthcare workforce. The program encompassed a national task force, stakeholder engagement initiatives, local conferences, and both regional and national policy summits. The task force comprised nurse leaders, PNAA representatives, regional chapter leaders, subject-matter experts, and other stakeholders involved in recruitment, onboarding, and workforce integration of IENs.

### 2.3. Sources of Data

This program evaluation utilized multiple data sources. Included were task force governance documents, program planning records, meeting notes, policy summit materials, local conference materials, aggregated survey findings from IENs, PNAA Human Rights Committee resources, and the *Handbook for Filipino Nurses Immigrating to the United States*.

These materials were utilized to outline the initiative’s structure, delineate key program activities, and summarize the primary policy and practice issues encountered during implementation. All data were reviewed in aggregate form. No individual-level identifying information was used.

### 2.4. Survey Data

Aggregated survey data from IENs were used to help identify program priorities. The survey asked about recruitment, pre-arrival preparation, onboarding, transition support, legal and administrative concerns, and workforce integration.

The survey included 232 respondents and was shared through professional networks and organizational partners involved in IEN workforce initiatives. Data were collected from April to June 2024 using a convenience sampling approach. Because the survey was used for program development and stakeholder engagement, response rates were not formally tracked.

Survey findings were reviewed descriptively and used to identify common gaps in recruitment, onboarding, and transition support. These findings helped guide policy summit discussions and informed the policy domains discussed in this paper.

### 2.5. Policy Summit Activities

Policy summits were held to bring together key stakeholders, including IENs, nurse leaders, healthcare organizations, recruitment agencies, legal experts, policymakers, and professional nursing organizations. The summits included presentations, panel discussions, and facilitated group sessions.

Group discussions used an appreciative inquiry approach. This approach focused on identifying what was working well, what challenges remained, and what practical strategies could strengthen IEN recruitment, onboarding, transition, and retention.

### 2.6. Data Analysis

A descriptive narrative synthesis was used to review the program materials. First, the documents were reviewed to identify the main components of the initiative, such as governance structure, task force roles, summit activities, and program outputs. We then identified common themes from the summit materials and discussion—summaries, including ethical recruitment, onboarding, transition support, workforce integration, and policy coordination.

Findings from different sources were compared to identify which issues recurred. Issues that appeared across more than one source were grouped into broader policy domains. The process helped to identify the key priorities for the initiative.

### 2.7. Trustworthiness

Several steps were used to support trustworthiness. First, multiple data sources were reviewed, including program documents, survey findings, summit materials, and program records. The sources were reviewed together to determine whether comparable problems were present across the different materials.

Second, the results were examined by members of the project team who were knowledgeable about the PNAA Cy Pres Project. This helped validate that the summary accurately reflected the program’s activities and deliverables.

Third, when the writers interpreted the findings differently, their interpretations were debated until a consensus was reached. The team reflected on whether the interpretation was compatible with the available facts, the program context, and the key priorities indicated across sources.

Together, these methods helped guarantee that the conclusions were not dependent on a single source and that the final summary was consistent with the overall program record.

### 2.8. Ethical Considerations

The PNAA Research Committee reviewed the project and determined that formal Institutional Review Board approval was not required. The evaluation used de-identified and aggregated program data. The survey was anonymous, did not collect sensitive information, and posed minimal risk to participants. All materials were handled in accordance with confidentiality and responsible data use.

## 3. Results

The PNAA Cy Pres program was established in 2024 to address challenges related to ethical recruitment, onboarding, transition support, and workforce integration of IENs. The initiative was designed as a national, organization-led effort. Important deliverables included a formal task force framework, stakeholder engagement activities, policy summits, and identification of important policy and practice priorities.

### 3.1. Governance Structure

The endeavor was a coordinated effort of the PNAA Human Rights Committee and the PNAA Cy Pres Task Force. The task force had a steering committee, a workgroup on program development, regional representatives, and external stakeholders, each with its own responsibilities (Table 1).

This structure provided clarity on roles, duties, and methods of communication. The steering group offered broad advice and helped ensure the program was aligned with PNAA’s aims. Program development workgroup: Planning activities, arranging summits, and stakeholder engagement. In the work, local and regional viewpoints were brought in by the regional delegates. Stakeholders provided input on recruitment, onboarding, transition support, and workforce integration.

### 3.2. Program Timeline and Key Activities

**Figure 1:**
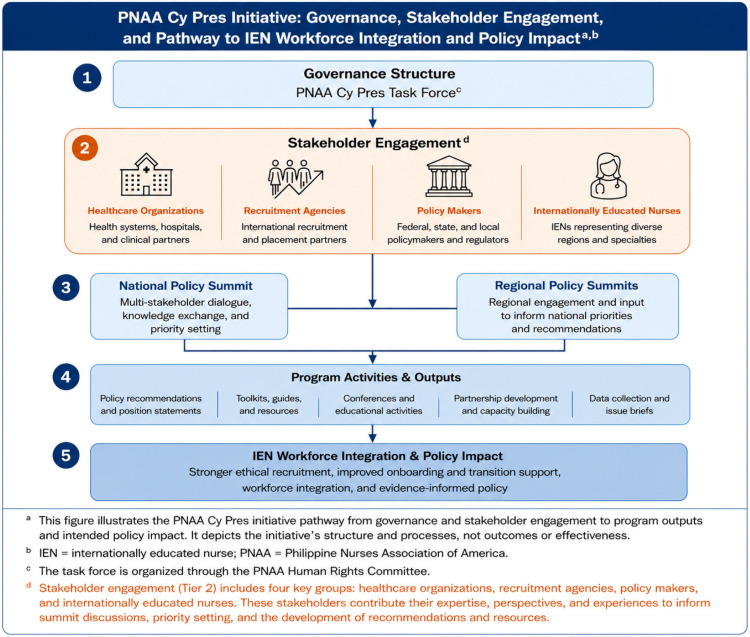
PNAA Cy Pres Initiative.

The program was created in stages. Early actions included organizing the task force, holding initial planning sessions, and constructing the program framework. This was followed by governance-related actions (e.g., defining roles and workgroup functions). Program planning thereafter centered on priority setting, development of summit objectives, and coordination of national and regional activities (Figure 1).

Local conferences and regional and national policy summits were also part of the initiative. These activities were leveraged to increase engagement and solicit input from a variety of stakeholders. Ongoing engagement was maintained through monthly meetings, stakeholder collaboration, and revision of program priorities.

### 3.3. Stakeholder Engagement and Policy Summit Activities

Policy summits were at the heart of the effort. The summits brought together IENs, nurse leaders, healthcare organizations, recruiters, legal experts, policymakers, and professional nursing organizations. The summit consisted of keynote lectures, panel discussions, and facilitated group sessions.

The group sessions were based on an appreciative inquiry method. Participants were asked to discuss what was working well, what barriers remained, and what approaches could improve IEN recruiting, onboarding, transition, and retention. This method allowed stakeholders to identify gaps and successful practices.

Across summit conversations and related program documents, stakeholders observed that IEN transition often engages a range of groups. Recruitment, legal advice, onboarding, coaching, and workplace integration may be handled individually. When these activities are not well coordinated, the transition process can become more difficult for both IENs and the organizations that employ them.

### 3.4. Policy and Practice Priorities Identified

Several policy and practice priorities were identified throughout program papers, summit talks, and survey-driven priorities (Table 2). These covered ethical recruitment, pre-departure preparation, onboarding and transition support, workforce integration, and policy governance.

The concerns expressed included ethical recruitment, transparency in contracts, financial burden, and accountability of recruitment partners. Preparation before leaving was also critical, as IENs may enter the U.S. workforce with varying degrees of readiness related to the health care system, job expectations, and administrative procedures.

Stakeholders consistently identified onboarding and transition support as important priorities. Stakeholders emphasized the need for comprehensive orientation, mentorship, cultural preparation, and support for the clinical and non-clinical aspects of transition. Workforce integration concerns included role adjustment, retention, and the need for coordinated support. Policy governance was highlighted as vital because many transition challenges require coordination among institutions, professional groups, recruitment agencies, and policymakers.

### 3.5. Implementation Model

**Figure 2:**
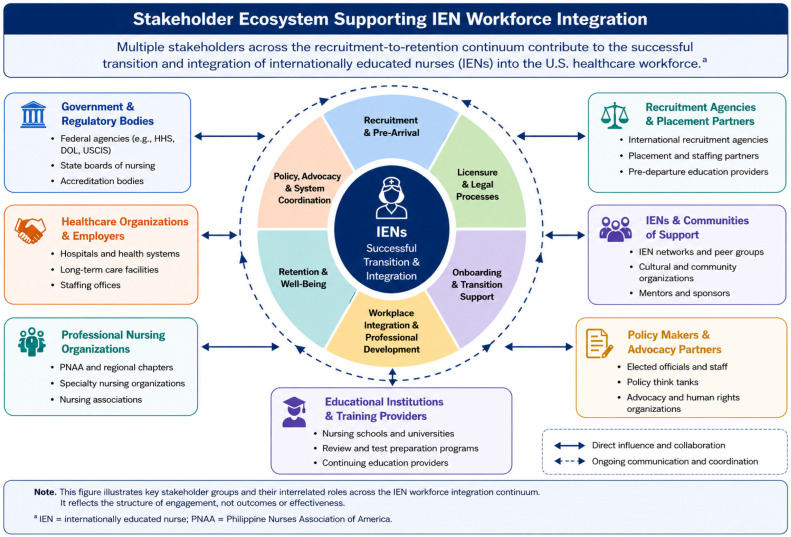
Stakeholder Ecosystem Supporting IEN Workforce Integration.

Findings from program evaluation were synthesized into a PNAA Cy Pres implementation model. The model highlights the linkages between governance, stakeholder involvement, policy summits, and the program’s deliverables (Figure 2). It also demonstrates that IEN workforce integration is not a one-time education or one-off onboarding activity. It is a coordinated approach to recruitment, preparation, onboarding, legal counsel, mentorship, and retention support.

The PNAA Cy Pres initiative generated multiple implementation deliverables, including a national governance structure, stakeholder engagement processes, activities from a policy summit, defined policy domains, and an implementation-informed model for future IEN workforce integration efforts. These outputs should be considered descriptive program findings, not proof of program efficacy or influence on the workforce.

## 4. Discussion

This paper describes the development and implementation of the PNAA Cy Pres initiative, a national effort to support IENs in entering and transitioning into the U.S. healthcare industry. The results show that the project developed a governance framework, involved a wide range of stakeholders, and prioritized various issues related to ethical recruitment, onboarding, workforce integration, and retention. Taken together, these actions demonstrate how a professional nursing association can help translate workforce policy ideals into practice.

One of the most valuable findings was stakeholder participation. The project involved healthcare organizations, recruitment agencies, policymakers, legal experts, professional nursing groups, and IENs. Previous research demonstrates that many of the issues faced by IENs stem from the distinct treatment of recruitment, onboarding, legal assistance, and workplace support, rather than from a lack of coordinated efforts [10,11]. The PNAA Cy Pres program was an attempt to address this fragmentation by creating chances for communication, collaboration, and shared problem-solving across sectors.

The findings also point to ongoing concerns about ethical recruitment and nurse mobility. Many high-income countries rely on international recruitment to help address nursing shortages. However, issues remain, including ambiguous contracts, recruiting expenses, power imbalances between nurses and employers or agencies, and protection of migrant nurses during recruitment and transfer [4,7,8,9]. These challenges are addressed by the PNAA Cy Pres program and its principles, which call for better transparency, accountability, and protection for workers.

A further important finding was the need for better onboarding and transition supports. Although many healthcare organizations invest significant effort and resources in recruiting internationally educated nurses, post-arrival support has been inconsistent [12,14,15]. Prior research has shown that mentorship, structured onboarding, cultural orientation, and professional supports can improve IENs’ integration and retention in the workforce [12,14,15]. Stakeholders at the policy summits identified the same needs, suggesting that these challenges remain common in practice.

The findings also show that professional nursing organizations can play an important role in integrating the IEN workforce. Much of the literature focuses on the individual nurse or the employer. Less attention has been given to how professional organizations can bring people together, advocate for change, and help turn workforce policy into action. Through policy summits and stakeholder engagement, the PNAA Cy Pres initiative offered one way to connect policy discussions with practical strategies. Although the initiative was developed within the Filipino nursing community, many of the issues identified also apply to IENs from other backgrounds.

The initiative illustrates how governance structures, stakeholder engagement, and collaborative problem-solving can help move workforce priorities into coordinated action. Appreciative inquiry helped the participants to see what they were already doing well and to work through their current challenges. This strengths-based approach is consistent with implementation-driven strategies that focus on pragmatic, solution-oriented approaches to workforce integration and retention challenges rather than barriers [16,17,18].

The findings also show that increasing the workforce is more than just hiring. It is about pre-hire preparation, post-hire onboarding, additional coaching, workplace support, and career advancement opportunities. Addressing these issues will require collaboration between health care facilities, recruitment agencies, professional nursing organizations, regulators, and legislators. This program has created a stakeholder ecosystem that illustrates the interrelationships among these roles and the need for shared accountability to support internationally educated nurses.

### 4.1. Implications for Practice and Policy

The findings point to a few practical steps. Healthcare organizations that hire IENs may need to look more closely at what happens after recruitment. Orientation should include clinical expectations, workplace culture, documentation, communication norms, and role adjustment.

Clear information also needs to be available early from recruitment agencies and employers. This includes contract terms, estimated fees, licensing requirements, job responsibilities, and the assistance provided. Better information could help to lessen confusion and stress during transition.

Also, part of the process should be mentorship. Mentors, peer support, and check-ins after the first few weeks of employment can be helpful for IENs. These services help nurses feel less isolated and more confident as they transition.

Professional nursing organizations can also assist by offering venues. The PNAA Cy Pres project is one example of a forum for IENs, their employers, recruiters, legal experts, and policy makers to discuss common issues and potential solutions.

### 4.2. Strengths and Limitations

The study had several strengths. It describes a national effort that engaged a wide range of stakeholders across international education networks, health care institutions, recruitment agencies, professional nursing societies, legal experts, and policy officials. It comprised a mix of information sources, including program documents, summit materials, survey findings, and stakeholder engagement. Taken together, these sources provide a full picture of issues related to IENs’ recruitment, onboarding, and integration into the workforce.

This study also has some limitations. This was a descriptive program evaluation, rather than an evaluation of effectiveness. As such, metrics such as retention, job satisfaction, onboarding success, and patient outcomes were not assessed. Second, a large portion of this material came from program documents and discussions with stakeholders and may reflect the views of project participants. Third, the findings may not apply to all internationally educated nurses or to all healthcare settings. Other actions and recommendations are not sufficiently evaluated, as they are still under development.

Despite these limitations, this study adds to the body of knowledge on how a professional nursing organization can facilitate IEN workforce integration through stakeholder engagement, policy dialogue, and collaborative planning. Future research should examine the effect of these interventions on workforce outcomes and the generalizability of these strategies to other settings and populations.

## 5. Conclusions

The PNAA Cy Pres initiative brought people together to talk about the needs of internationally educated nurses. The initiative helped identify common concerns about recruitment, onboarding, transition support, and workforce integration. More evaluation is still needed, but this initiative offers a useful starting point for improving the support IENs receive when they enter the U.S. healthcare workforce.

## Figures and Tables

**Table 1:** PNAA Cy Pres Task Force Structure and Responsibilities.

Component	Description	Key Responsibilities	Stakeholders Involved
**Steering** **Committee**	Provides overall strategic direction and oversight of the initiative	Align program with PNAA priorities; guide policy direction; oversee resource allocation	PNAA leadership, senior advisors
**Program** **Development Workgroup**	Leads operational planning and implementation activities	Develop program strategies; coordinate summits; manage stakeholder engagement	Nurse leaders, subject matter experts, and regional representatives
**PNAA Human Rights** **Committee**	Serves as the oversight body ensuring alignment with ethical recruitment and workforce advocacy goals	Monitor ethical standards; ensure alignment with PNAA mission; support policy advocacy.	PNAA executive leadership, policy advisors
**Regional** **Representatives**	Facilitate regional engagement and implementation of initiative activities.	Coordinate regional summits, engage local stakeholders, and provide contextual insights.	Regional PNAA chapters, local leaders
**External** **Partners and** **Stakeholders**	Contribute expertise and participate in policy discussions and implementation activities.	Provide input on recruitment, onboarding, and workforce integration; participate in summits.	Healthcare organizations, recruitment agencies, policymakers, and IENs

**Table 2:** Key Policy Domains, Challenges, PNAA Cy Pres Strategic Responses and Policy Implications.

Policy Domain	Key Challenges Identified	PNAA Cy Pres Strategic Responses	Policy Implications
**Ethical Recruitment**	Lack of transparency; inconsistent contracts; financial burden on IENs	Stakeholder dialogues with recruitment agencies; promotion of ethical recruitment standards	Need for standardized and enforceable ethical recruitment guidelines
**Pre-Departure Preparation**	Variable training; limited system orientation; inconsistent expectations	Identification of best practices through summits; emphasis on pre-arrival readiness	Development of standardized pre-departure training frameworks
**Onboarding & Transition**	Inconsistent onboarding; limited cultural orientation; fragmented support	Promotion of structured onboarding models, mentorship, and integration strategies	Adoption of comprehensive onboarding programs across institutions
**Workforce Integration**	Role adaptation challenges, administrative barriers, and a lack of coordinated support	Cross-sector collaboration; alignment of organizational practices	Integration of system-level coordination across healthcare organizations
**Policy & Governance**	Fragmented policies; lack of coordinated oversight	National and regional policy summits; stakeholder engagement	Strengthening multi-level governance and workforce policy alignment

## Data Availability

The datasets generated and/or analyzed in the current study are not publicly available due to data governance and organizational considerations but may be made available from the corresponding author upon reasonable request and with permission from the PNAA Research Committee.
